# A new species of *Hyphessobrycon* (Characiformes, Characidae) from the upper Guaviare River, Orinoco River Basin, Colombia

**DOI:** 10.3897/zookeys.668.11489

**Published:** 2017-04-13

**Authors:** Carlos A. García-Alzate, Alexander Urbano-Bonilla, Donald C. Taphorn

**Affiliations:** 1 Universidad del Atlántico, Programa de Biología, Barranquilla, Colombia; 2 Laboratorio de Ictiología, Departamento de Biología, Facultad de Ciencias, Pontificia Universidad Javeriana, Carrera 7 N° 43-82, Bogotá, D.C., Colombia; 3 1822 North Charles Street, Belleville, Illinois, 62221, USA

**Keywords:** New taxon, Neotropical Ichthyology, Guaviare River, diversity, Nuevo taxón, Ictiología Neotropical, río Guaviare, diversidad

## Abstract

*Hyphessobrycon klausanni* **sp. n.** is described from small drainages of the upper Guaviare River (Orinoco River Basin) in Colombia. It differs from all congeners by having a wide, conspicuous, dark lateral stripe extending from the anterior margin of the eye across the body and continued through the middle caudal-fin rays, and that covers (vertically) three or four horizontal scale rows. It also differs by having an orange-yellow stripe extending from the anterosuperior margin of the eye to the caudal peduncle above the lateral line in life. It differs from all other species of *Hyphessobrycon* that have a similar dark lateral stripe: *H. cyanotaenia*, *H. loretoensis*, *H. melanostichos*, *H. nigricinctus*, *H. herbertaxelrodi*, *H. eschwartzae*, *H. montogoi*, *H. psittacus*, *H. metae*, *H. margitae*, *H. vanzolinii*, and *H. peruvianus* in having only three or four pored scales in the lateral line, 21 to 24 lateral scales and six teeth in the inner premaxillary row. *Hyphessobrycon klausanni* differs from *H. loretoensis* in having seven to eight maxillary teeth (vs. three to four) and in having a longer caudal peduncle (12.4–17.0% SL vs. 4.6–8.0% SL). Additionally *Hyphessobrycon klausanni* can be distinguished from the other species of *Hyphessobrycon* with a dark lateral stripe from the Orinoco River Basin (*H. metae* and *H. acaciae*) in having two teeth in the outer premaxillary row (vs. three to four) and 10 branched pectoral–fin rays (vs. 11 to 12). It further differs from *H. metae* by the length of the snout (17.6–22.8% HL vs. 9.9–15.2% HL) and by the length of the caudal peduncle (12.4–17.0% SL vs. 7.3–11.8% SL).

## Abstract (es)

*Hyphessobrycon klausanni* **sp. n.** es descrita de pequeños drenajes del alto río Guaviare (cuenca del río Orinoco) en Colombia. Se diferencia de todos los congéneres por tener una amplia franja lateral oscura lateral que se extiende desde el margen anterior del ojo a través del cuerpo y se continúa hasta la base de la aleta caudal y que cubre (verticalmente) tres o cuatro escamas laterales, además difiere por tener una franja de color naranja-amarillo que se extiende desde el margen anterosuperior del ojo hasta el pedúnculo caudal por encima de la línea lateral en vida. Se diferencia de *H. cyanotaenia*, *H. loretoensis*, *H. melanostichos*, *H. nigricinctus*, *H. herbertaxelrodi*, *H. eschwartzae*, *H. montogoi*, *H. psittacus*, *H. metae*, *H. margitae*, *H. vanzolinii*, y *H. peruvianus* por tener solo 3 a 4 escamas con poros en la línea lateral, 21 a 24 escamas laterales y 6 dientes en la fila interna del premaxilar. *Hyphessobrycon klausanni* se diferencia de *H. loretoensis* por presentar 7 a 8 dientes maxilares (vs. 3 a 4) y la longitud del pedúnculo caudal (12.4–17.0% LE vs. 4.6–8.0% LE). Además se puede distinguir de las demás especies con banda lateral oscura presentes en la cuenca del rio Orinoco (*H. metae* y *H. acaciae*) por tener dos dientes en la fila externa del premaxilar (vs. 3 a 4) y 10 radios ramificados en la aleta pectoral (vs. 11 a 12), además se separa de *H. metae* por la longitud de hocico (17.6–22.8% LC vs. 9.9–15.2% LC) y por la longitud del pedúnculo caudal (12.4–17.0% LE vs. 7.3–11.8% LE).

## Introduction

*Hyphessobrycon* Durbin, 1908, with 147 valid species (Eschmeyer et al. 2016), is a member of the subfamily Tetragonopterinae in Characidae (Mirande 2010). In addition to being one of the largest characid genera, it is found in all major drainages of the Neotropics, from southern México to the La Plata River in Argentina (García-Alzate et al. 2013а). In morphological and molecular phylogenetic analyses Mirande (2010) and Oliveira et al. (2011) respectively, state that *Hyphessobrycon* is paraphyletic. The genus was proposed by Durbin in Eigenmann (1908) as a subgenus of *Hemigrammus*, the type species of which is *Hemigrammus unilineatus* (Gill, 1858) described from the Island of Trinidad. *Hyphessobrycon* is defined by the following combination of non-exclusive characters: lateral line incompletely pored, adipose fin present, few or no teeth present on the maxilla, third infraorbital bone not in contact with the preopercle, premaxilla with two rows of teeth, with five or more on each side of the inner row, and caudal fin not covered with scales (the character that supposedly differentiates it from *Hemigrammus*). Within *Hyphessobrycon*, species have been grouped primarily by similarities of colour pattern; some of which were proposed merely as artificial operational assemblages to aid species identification (Géry 1977), whereas others represent potential monophyletic groups, as is the case for the Rosy Tetra species group (Castro Paz et al. 2014).

Twenty-two species of *Hyphessobrycon* have been identified from the different hydrographic drainages in Colombia (García-Alzate et al. 2015) and thirteen species are found in streams of the Orinoco River Basin: *H. acaciae*, *H. albolineatum*, *H. bentosi*, *H. diancistrus*, *H. epicharis*, *H. fernandezi*, *H. heterorhabdus*, *H. mavro*, *H. metae*, *H. niger*, *H. otrynus*, *H. sweglesi*, and *H. saizi*. The objective of this paper is to describe a new species of *Hyphessobrycon* from the upper Guaviare River drainage, which is part of the Orinoco River Basin in Colombia.

## Materials and methods

Fishes were captured using seines and were preserved in situ in 10% formalin and later stored in 70% ethanol. Counts and measurements follow Fink and Weitzman (1974). Measurements were made with digital callipers to 0.1mm precision and are expressed as percentages of standard (**SL**) and head length (**HL**). In count ranges, values for the holotype are indicated with an asterisk (*) and number of individuals after the meristic counts in parentheses. Counts and measurements were taken on the left side of specimens when possible. Osteological observations were made on cleared and stained adult specimens (**CS**) prepared according to Taylor and Van Dyke (1985). Bone nomenclature follows Weitzman (1962) and Vari (1995). Type specimens are deposited in the University of Atlántico-Caribbean Region, Dept. Biology, Museum Collection, Barranquilla, Colombia (**UARC-IC**), Auburn University Natural History Museum Fish Collection, Auburn, Alabama (**AUM**), the Ichthyology Laboratory at the Universidad del Quindío, Armenia, Colombia (**IUQ**) and Museo Javeriano de Historia Natural “Lorenzo Uribe, S. J.”, Bogotá D. C. (**MPUJ**). In the list of paratypes, the number of individuals is given in parentheses immediately after the catalog number. Institutional abbreviations are as listed at ttp://www.asih.org/node/204, except UARC-IC. We performed a Principal Components Analysis (**PCA**) of morphometric characters of *Hyphessobrycon metae*, *H. acaciae*, *H. mavro*, *H. niger*, and *H. klausanni* and the Burnaby method was used to eliminate the influence of overall size, with the Past program, version 3.0 for Windows (Hammer et al. 2001). Species for which no specimens were available, such as *Hyphessobrycon montogoi*, *H. margitae*, *H. vanzolinii*, *H. lucenorum*, *H. psittacus*, and *H. peruvianus* were included in the comparisons using their original descriptions. The abbreviation masl means meters above sea level.

### Comparative material examined

***Hyphessobrycon acaciae***: COLOMBIA, Meta: IUQ 2796, holotype, Morichal del Estero, Puerto López, Meta, ~ 4°04'N; 72°57'W, 10 Sep. 1994. Paratypes: MPUJ 393, 28, collected with holotype; IUQ 2795, 2 CS, collected with holotype; IUQ 2433, 49, Laguna Hacienda La Cabaña, Inspección de Surinera, San Carlos, Acacías, ~03°55'N; 73°50'W, 7 Jan. 2009; IUQ 2492, 17, Acacías Creek on road to Vista Hermosa-Puerto Lucas, 03°06'51"N; 73°45'44"W, 259 masl, 08 Jan. 2009; IUQ 2793, 4 CS, Laguna Hacienda La Cabaña, Inspección de Surinera, San Carlos, Acacías, 07 Jan. 2009. MPUJ 2604, 52, Laguna El Retiro, Inspección La Loma, Acacías, 11 Oct. 2006. ***H. metae***: Colombia, Meta: CAS 61751, Holotype, Río Meta in Barrigona, Orinoco River drainage, 1914; IAvH-P 3014, 57, Caño Muco, Hacienda San Francisco, Puerto Gaitán-Gaviotas road, 3°13'49"N; 73°52'39"W; IavH-P 6151, Vichada, Las Galapagitas Creek near camp in La Sabana, Puerto Carreño, Vichada, 30 Apr. 1990. Venezuela, Guárico: MCNG 32469, 19, Aguaro Guariquito National Park, San José River, 13 Jan. 1995. ***H. mavro***: Colombia, Vichada: IUQ 2791, Holotype, Payara Creek, tributary of Negro Creek, Puerto Carreño, ~6°12'N; 67°28'W, 27 Apr. 2005; IMCN 3751, 21, Paratypes, collected with holotype; IUQ 1964, 2 CS, collected with holotype. ***H. niger***: Colombia, Meta: IUQ 2792, holotype, Mojaculo Creek, Vereda Dinamarca, Acacías, Meta, 03°53'20.6"N; 73°28'30"W, 03 Apr. 2008; MPUJ 5039, 28, Paratypes, collected with holotype; IUQ 2794, 2 CS, collected with holotype. ***H. agulha***: Colombia, Amazonas, Leticia: IAvH-P 8345, 19, tributary of Matamata Creek Mar. 2001; IAvH-P 8335, 37, tributary of Matamata Creek, 18 Mar 2001; IAvH-P 8332, 4, tributary of Matamata Creek, 02 Jul 2001; IAvH-P 8333, 52, tributary of Purité River, 25 Mar 2001; IAvH-P 9025, 85, Sufragio in front of Zafire Station, 15 Dec 2002; IAvH-P 9046, 14, stream, tributary of Calderón River, 45 minutes north of Zafire Station, 11 Dec 2002; IAvH-P 9071, 38, tributary of Calderón River, 45 min. north of Zafire Station, 12 Dec 2002; IAvH-P 9407, 25, stream 2, tributary of Purité River three hours from Salados Varios, Amacayacu National Park, 25 Ma. 2003; Perú, Madre de Dios: MUSM 23173, 9, stream at km 43 Tambopata; MUSM 23173, 1 CS, stream at km 43 Tambopata; MUSM 25315, 9, stream at km 43 Tambopata; MUSM 25315, 1 CS, stream at km 43 Tambopata. ***H. compressus***: México: FMNH 4641, holotype, Obispo, Vera Cruz; FMNH 4662, 1 CS paratypes, collected with holotype; BMNH 1905.12.6.4-5, 2 paratypes, Obispo, Vera Cruz; IBUAM-P 8538, 2, Trinitaria, Flor de Café, Chris, 3 Jul. 1993; ANSP 124774, 12, Río Usumacinta almost connected to Pasión, near Sayache, 18 Aug. 1996; ANSP 124774, 3 CS, Río Usumacinta almost connected to Pasión, near Sayache, 18 Aug. 1961. ***H. herbertaxelrodi***: Brazil: MCP 30829, 5, stream tributary to the Caibi River, at its mouth, Mato Groso, 18 Jan 2002; MCP 30829, 2 CS, Brazil, stream tributary to the Caibi River, at its mouth, Mato Grosso, 18 Jan 2002. ***H. heterorhabdus***: CAS 44415, Syntype, 1, 16.9 mm de SL, Brazil, Para, 1894; ICNMNH 5063, 10, 17.8-23.9 mm SL, Colombia, Amazonas, Puré River, Leticia 02° 07'05"S; 69°37'50"W, 8 Jan 2000; MCP 41577, 5, 19.5-23.6 mm SL, Brazil, Para, Igarapé Acuí, 01°35'46"S; 48°44'26"W, 21 Oct 2006; IUQ 1961, 3 CS, 28.3-34.6 mm SL, Colombia, Puré River, Leticia, Amazonas, 02°07'05'S; 69°37'50"W, 8 Jan 2000; IUQ 1963, 1 CS, 33.1 mm SL, Brazil, Para, Igarapé Acuí Igarapçe Acuí, 01°35'46"S; 48°44'26"W. ***H. loretoensis***: MUSM 20179, 2, Perú, Loreto, upper Amazon, Morona River drainage, Anazo Creek, 7 May 2002; MUSM 23172, 4, Perú, Loreto, upper Amazon, Abanico del Pastaza, Corrientes River drainage, Platanoyacu River, 27 Oct 2004; MUSM 23233, 5, 20.7-30.2 mm SL, Perú, Loreto, upper Amazon, Abanico del Pastaza, Corrientes River drainage, Platanoyacu River, 27 Oct 2004. ***H. melanostichos***: MCP 39808, 5, Brazil, Doze de Outubro River between Comodro and Vilhena, 12°35'46"S; 60°00'30"W, 14 Jul 2004. ***H. nigricinctus***: MUSM 26791, 1, 32.04 mm SL, Perú, Cusco, Quispicanchi, Camanti Cuenca Araza, San Lorenzo River, Ilahuala Creek, 26 Oct 2005; MUSM 26786, 5, Perú, Cusco, Quispicanchi, Camanti, Araza River drainage, San Lorenzo River, Ilahuala Creek, 26 Oct 2005. ***H. notidanus***: MCP 38676, 2 paratypes, 24.8-25.7mm SL, Brazil, Doze de Outubro River between Comodoro and Vilhena, 12°58'39"S; 60°00'30"W, 14 Jul 2004; MCP 38676, 1 CS, Brazil, Doze de Outubro River between Comodoro and Vilhena, 12°58'39"S; 60°00'30"W, 14 Jul 2004. ***H. taphorni***: Perú, Madre de Dios: MUSM 42391, holotype, Paiche pool, Aguajal Baja, Madre de Dios, Tambopata River, 12°29'4.2"S; 68°57'9.09"W, 22 Feb 2004; MUSM 22042, 95, paratype, collected with holotype; AUM 56757, 2 paratypes, Aguajal de Aguas Negras, mining pond, Tambopata River, Madre de Dios River drainage, 12°38'9.84’’ S; 69°25'35.8"W, 21 Jan 2004; AUM 56758, 2 paratypes, Aguajal Este, Tambopata River, Madre de Dios River drainage, 20 Feb. 2004; MUSM 5562, 60 paratypes; Sandoval Lake, Las Piedras, Tambopata River drainage, 12°36'17.76"S; 69°02'40.2"W, 23 Jan. 1990; MUSM 5581, 4 paratypes, Sandoval Lagoon, Las Piedras, Tambopata River drainage, 12°36'17.76’’ S; 69°02'40.29"W, 24 Jan 1990; MUSM 5588, 80 paratypes, Tambopata, Las Piedras, Quebrada 2 km from Sandoval Lagoon, 12° 36'17"S; 69°03'23"W, 23 Jan.1990; MUSM 9584, 11 paratypes, Sandoval Lake, Tambopata, Madre de Dios River drainage, 12°36'17.76"S; 69°02'40.29"W, 15 May. 1996; MUSM 21703, 15 paratypes, Aguajal Aguas Negras, Aguajal Satélite, Tambopata River, Madre de Dios River drainage, 12°39'22"S; 69°26'28"W, 22 Jan. 2004; MUSM 21824, 58 paratypes, Aguajal de Aguas Negras, mining pond, Tambopata River, Madre de Dios River drainage, 12°38'9.84"S; 69°25'35.8"W, 21 Jan 2004; MUSM 21994, 272 paratypes, Aguajal Este, Tambopata River, Madre de Dios River drainage, 20 Feb. 2004; IUQ 3032, 2 CS paratypes; Aguajal Aguas Negras, Agujal Satélite, Tambopata River, Madre de Dios River drainage, 12°39'22"S; 69°26'28"W, 22 Jan. 2004. ***H. eschwartzae***: Perú, Madre de Dios: MUSM 42392, holotype, La Cachuela, Madre de Dios River, Tambopata, 12°16'38.2"S; 69°09'8.12"W, 8 Jul. 2003; AUM 51350, 12 paratypes, Río Planchón, crossing the bridge of the Interoceanic highway, 36.3 km N of Puerto Maldonado, Río Inambari, 01 Aug. 2010; AUM 51374, 16 paratypes, Río Buyuyoc, at bridge of Interoceanic highway, 91.1km N of Puerto Maldonado, 01 Aug. 2010; MUSM 22474, 111 paratypes, collected with holotype; MUSM 3684, 16 paratypes, Tambopata River, Quebrada 500 m from campsite, Sandia, Puno, 26 Aug. 1992; MUSM 9771, 26 paratypes, Madre de Dios River, km 29, Tambopata, 12°42'26.22"S; 69°26'54.03"W, 22 May. 1996; MUSM 21221, 143 paratypes, Quebrada at km 14 on road to San Juan, Manuripe Alegría River drainage, 12°0'57.41"S; 69°03'43.39"W; MUSM 22893, 30 paratypes; Quebrada El Planchón (km 40), Tambopata, 12°16'34.94"S; 69°09'8.12"W, 13 Jul, 2003; IUQ 3033, 3 CS paratypes, Quebrada at km 14, road to San Juan, Manuripe Alegría River drainage, 11 Jul 2003. ***H. oritoensis***: Colombia, Putumayo, Orito: IUQ 1574, Holotype, Quebrada La Palma, La Palma ranch, Vereda Calimonte, 29 Jun. 1998, IUQ 139, 6 Paratypes, collected with the holotype. IUQ 1575, 2 CS, paratypes, collected with the holotype; MBUCV-V 33737, 2 paratypes, collected with the holotype; MCNG 55844, 2, paratypes, collected with the holotype. ***H. paucilepis***: Venezuela, Lara state: IUQ 1897, Holotype, Los Quediches Reservoir, overflow channel, 03 Sep. 1987; MBUCV-V 23710, 3 paratypes, collected with holotype; IUQ 1898, 1 CS paratype, collected with holotype; MBUCV-V 903, 4 CS paratypes, Los Quediches Reservoir, overflow channel; MBUCV-V 23706, 42 paratypes, Los Quediches Reservoir, overflow channel, MBUCV-V 6933, 6 paratypes, Burere, Carora-Cabimas highway. CPUCLA 532, 5 paratypes, Ciénaga de Puricaure Carora - El Venado highway, about 1 km from Puricaure, Quebrada Arriba road, 10°06'26.0"N; 70°28'93"W. ***H. vilmae***: MCP 38881, 5, 28.0-30.6 mm SL, Brazil, Papagaio River in front of public beach, Mato Grosso 13°33'35"S; 58°24'31"W, 13 Jul 2004.

## Taxonomy

### Hyphessobrycon klausanni sp. n.

Taxon classificationAnimaliaCharaciformesCharacidae

http://zoobank.org/B648B8AE-BE21-44B9-A5A5-D2BD5CEF9EE9

**Figure 1.**
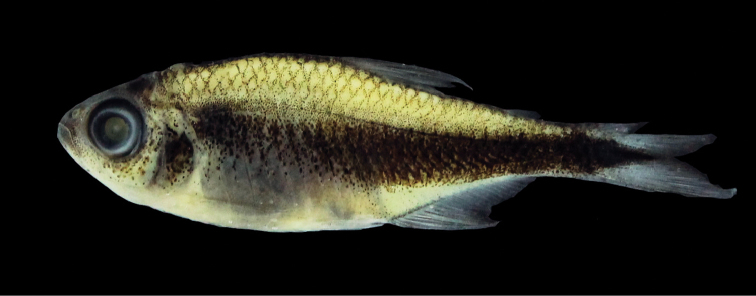
*Hyphessobrycon klausanni* sp. n., UARC-IC 539, holotype, male, 23.1 mm SL, Mapiripán County, upper Guaviare River drainage, Caño Claro, Meta, Colombia.

**Figure 2.**
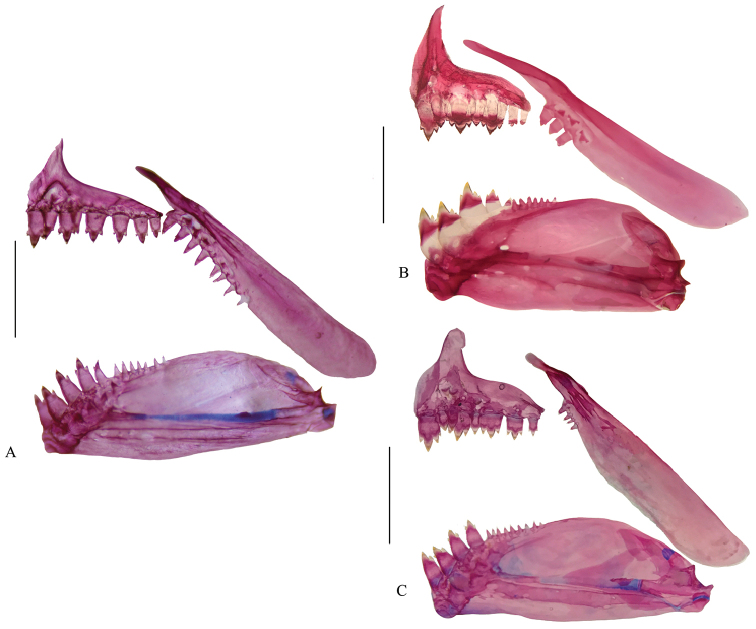
Upper and lower jaws of **A** *Hyphessobrycon klausanni* sp. n., UARC-IC 541, 20.2 mm SL **B** *H. metae* MCNG 32469, 19.9 mm SL and **C** *H. acaciae*, IUQ 2795 Paratypes, 28.6 mm SL. Scale bar: 1 mm.

**Figure 3.**
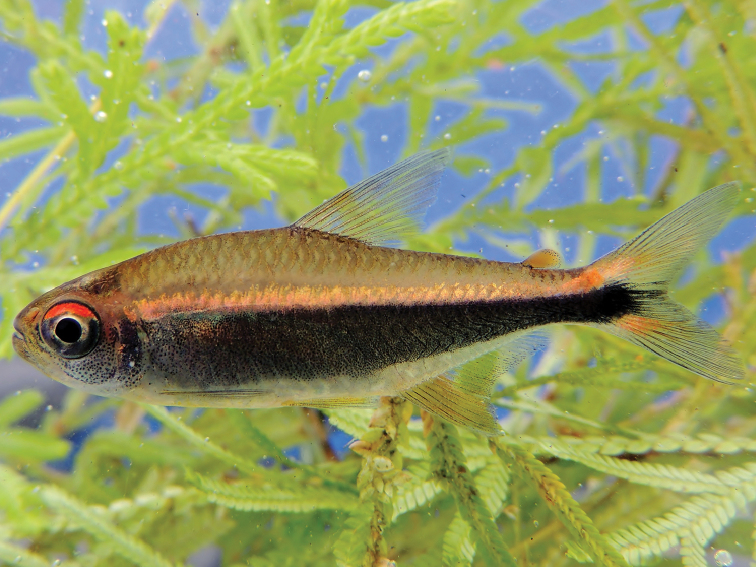
Live colours of *Hyphessobrycon klausanni* sp. n. Paratype, UARC–IC 540, 22.4 mm SL.

**Figure 4.**
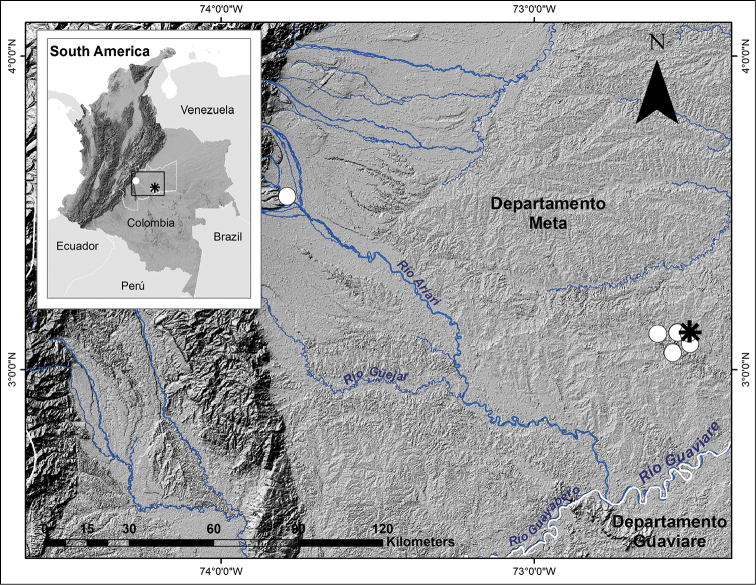
Distribution of *Hyphessobrycon klausanni* sp. n. in the upper tributaries of Guaviare River drainage. Type locality indicated by an asterisk.

Figures 1 , 2 , 3 , 4

#### Holotype.

UARC-IC 539, 23.1 mm SL, male, Colombia, Meta, Mapiripán County, upper Guaviare River drainage, Caño Claro, 03°07'05.1"N; 72°30'14.8"W; 209 masl; **Paratypes**. UARC-IC 540, five, 22.1–24.2 mm SL, collected with holotype; UARC-IC 541, two CS, 20.2–22.3 mm SL, collected with holotype; UARC-IC 542, eight, 20.1–23.3 mm SL, Mapiripán county, upper Guaviare River drainage, Caño La División, 03°07'05.8"N; 72°32'36.7"W, 209 masl; UARC-IC 543, two, 25.1–28.4 mm SL, El Castillo County, upper Guaviare River drainage, Caño Hondo, 03°33'08.6"N; 73°47'17.9"W, 209 masl; UARC-IC 544, six, 22.1–23.4 mm SL, Mapiripán County, upper Guaviare River drainage, Caño División 2, 03°07'03.3"N; 72°32'32.5"W, 221 masl; MPUJ 7857, eight, 25.4–31.4 mm SL, El Castillo County, Caño Hondo, upper drainage of Guaviare River, 03°33'08.6"N; 73°47'17.9"W.

#### Diagnosis.

*Hyphessobrycon klausanni* sp. n. can be diagnosed from all other species of *Hyphessobrycon*, except of *H. cyanotaenia*, *H. loretoensis*, *H. melanostichos*, *H. nigricinctus*, *H. herbertaxelrodi*, *H. eschwartzae*, *H. montogoi*, *H. psittacus*, *H. metae*, *H. margitae*, *H. vanzolinii* and *H. peruvianus*, by having a wide, conspicuous, dark lateral stripe extending from the anterior margin of the eye across the head and body and continuing through the middle caudal-fin rays to their tips. It differs from the species excepted above in having a wider lateral stripe that covers three or four horizontal scale rows (vs. stripe covering just one or two scales rows); in having an orange-yellow stripe extending from the anterodorsal margin of the eye to the caudal peduncle above the dark lateral stripe in life (vs. red lateral stripe extending from the anterodorsal margin of the eye to the caudal peduncle above the dark lateral stripe in live *H. heterorhabdus*, *H. amapaensis*, *H. eschwartzae* and *H. montagi*); by having fewer pored lateral line scales (three to four vs. five to10); fewer lateral scales (21 to 24 vs. 29 to 34); and in having more teeth on the inner premaxillary row (six vs. five). It differs from *H. loretoensis* in having seven to eight maxillary teeth (vs. three to four) and in having a longer caudal peduncle (12.4–17.0% SL vs. 4.6–8.0% SL). Additionally *Hyphessobrycon klausanni* can be distinguished from the other species of *Hyphessobrycon* with a dark lateral stripe from the Orinoco River Basin (*H. metae* and *H. acaciae*), in having: two teeth in the outer premaxillary row (vs. three to four see Fig. 2) and 10 branched pectoral-fin rays (vs. 11 to 12). It further differs from *H. metae* by the length of the snout (17.6–22.8% HL vs. 9.9–15.2% HL) and by the length of the caudal peduncle (12.4–17.0% SL vs. 7.3–11.8% SL).

#### Description.

Morphometric data shown in Table 1. Body compressed, moderately thick, greatest depth between pelvic-fin insertions and dorsal-fin origin. Dorsal profile of head straight from upper lip to vertical through middle of orbit; then convex to dorsal-fin origin. Dorsal-fin base convex, then slightly concave to adipose-fin origin, then straight to base of upper caudal-fin lobe. Ventral profile of head convex from lower lip to insertion of anal fin, anal-fin base straight and then slightly concave to base of lower caudal-fin lobe.

**Table 1.** Morphometric and meristic data of *Hyphessobrycon klausanni* sp. n. Standard length given in mm. Mean given in parentheses. SD = Standard deviation.

	Holotype	Paratypes	SD
Standard length	23.1	20.1–28.4 (22.2)	1.2
Total length	27.5	25.8–29.3 (27.5)	0.9
**Percentages of SL**			
Body depth	29.4	29.4–35.1 (32.5)	1.5
Snout-dorsal fin distance	50.2	49.7–56.5 (53.7)	2.5
Snout-pectoral fin distance	32.0	25.7–33.6 (30.2)	2.2
Snout-pelvic fin distance	44.2	42.8–53.7 (47.7)	3.5
Snout-anal fin distance	57.1	57.1–69.2 (62.0)	3.3
Dorsal fin-hypural distance	47.2	47.1–53.5 (50.4)	2.0
Dorsal-fin length	29.9	29.2–35.0 (31.9)	2.1
Pectoral-fin length	38.5	35.0–45.0 (39.2)	3.1
Pelvic-fin length	26.0	23.2–29.9 (25.9)	2.1
Caudal peduncle depth	21.2	17.5–24.5 (20.8)	2.2
Caudal peduncle length	15.2	15.1–20.9 (17.9)	1.8
Head length	22.5	20.3–25.3 (21.5)	1.3
Dorsal-anal fin distance	10.0	8.7–12.7 (10.0)	1.1
Dorsal-pectoral fin distance	15.2	12.2–17.0 (14.4)	1.2
Anal-fin length	28.6	27.2–31.3 (29.6)	1.5
**Percentages of HL**			
Snout length	18.2	17.6–22.8 (20.4)	1.8
Orbital diameter	36.4	30.1–45.0 (37.9)	4.9
Postorbital distance	39.4	36.6–53.1 (43.5)	4.3
Maxilla length	39.4	23.9–49.2 (35.6)	7.4
Interorbital distance	39.4	29.5–41.5 (25.8)	3.4
Mandible superior distance	27.3	22.5–30.4 (26.4)	2.5
**MERISTICS**			
Lateral scales	24	20–24	
Pored lateral-line scales	4	3–4	
Scales from lateral line to dorsal fin	5	5	
Scales from lateral line to anal fin	4	4	
Scales from lateral line to pelvic fin	3	3	
Predorsal scales	9	9–10	
Dorsal-fin rays	ii, 9	ii, 9	
Anal-fin rays	iii, 20	iii, 19–20	
Pelvic-fin rays	ii, 7	ii, 7	
Pectoral-fin rays	ii, 10	ii, 10	

Head and snout long, jaws equal, mouth terminal, lips soft and flexible, outer row of premaxillary teeth not exposed. Premaxilla with long, sharp lateral process over ethmoids, with two rows of teeth: the external row with one (2) or two* (23), all tricuspid; inner row with six* (25) tricuspid teeth the gradually diminish in size away from symphysis. Maxillary long and narrow, posterior margin straight, anterior margin convex, ventral margin reaching anterior border of third infraorbital, with seven* (10) or eight (15) tricuspid and conical teeth. Dentary with convex ventral margin, four (25) frontal multicuspid teeth followed by six* (15) or eight (10) smaller conical teeth (Fig. 2).

Scales cycloid. Lateral line with three (10) or four* (15) pored scales. Lateral scale series with 21 (10), 22 (6) or 24* (9) scales, including those with pores. Transverse scales rows five* (25) between dorsal-fin origin and lateral line, not including the predorsal series just in front of first dorsal-fin ray. Four (25) horizontal scale rows from anal-fin origin to lateral line. Three (25) horizontal scale rows between pelvic-fin origins and lateral line. Predorsal scales nine*(18) or 10 (7). Five scales in single row along anterior anal-fin base. Fin rays: Dorsal ii, 9 (25). Anal iii, 19 (18) or 20* (7). Pelvic ii, 7 (25). Pectoral ii, 10 (25). Caudal 10+10 (2) principal and 10 (2) procurrent. Caudal fin bifurcate, upper and lower lobes similar in size, pointed. Total vertebrae 32–33.

#### Sexual dimorphism.

Males have hooks on anal-fin and pelvic–fin rays. Anal fin with pair of rows of eight to ten small hooks along third simple ray followed by two to eight pairs of hooks on first to fifth branched rays. Pelvic fins with two to ten pairs of hooks on branched rays, located above internal branch of ray, each segment of branched rays with pair of hooks.

#### Color in alcohol.

Opercular and humeral spots absent. Dorsal part of head and body to dorsal fin dark brown, then yellow on sides and light yellow ventrally. Base color divided by conspicuous, wide (three to four horizontal scale rows), dark lateral stripe from anterior margin of eye through middle caudal-fin rays. Pectoral, pelvic and anal fins hyaline. Dorsal, anal and caudal fins with dark margins. Anal fin with dark pigment concentrated on interradial membranes.

#### Color in life.

Body greenish-yellow, predorsal area orange-yellow, preventral area silvery-yellow, upper and lower margins of eye red and black respectively, dorsal area of head orange-yellow, ventral area greenish-yellow with great concentration of melanophores on infraorbitals, preopercle and opercle. Wide, black, lateral stripe from anterior part of eye along sides through middle caudal-fin rays, covering at least half of body height near midbody. Iridescent orange stripe present above black stripe from eye to upper caudal-fin lobe. Lower lobe of caudal fin with orange iridophores at bases of rays. Bases of dorsal-fin rays orange, base of caudal and pelvic fins greenish–yellow. Adipose fin light orange (Fig. 3).

#### Distribution.

*Hyphessobrycon klausanni* is known only from the type locality in the upper Guaviare River Orinoco Basin in Colombia (Fig. 4).

#### Etymology.

Research leading to the discovery and recognition of this species was partially funded by Mr. Klaus-Peter Lang from Oberhausen, Germany. To commemorate the 80^th^ birthday of his mother, this species is dedicated to and named for his father “Klaus” and his mother “Anni”.

#### Remarks.

**Figure 5.**
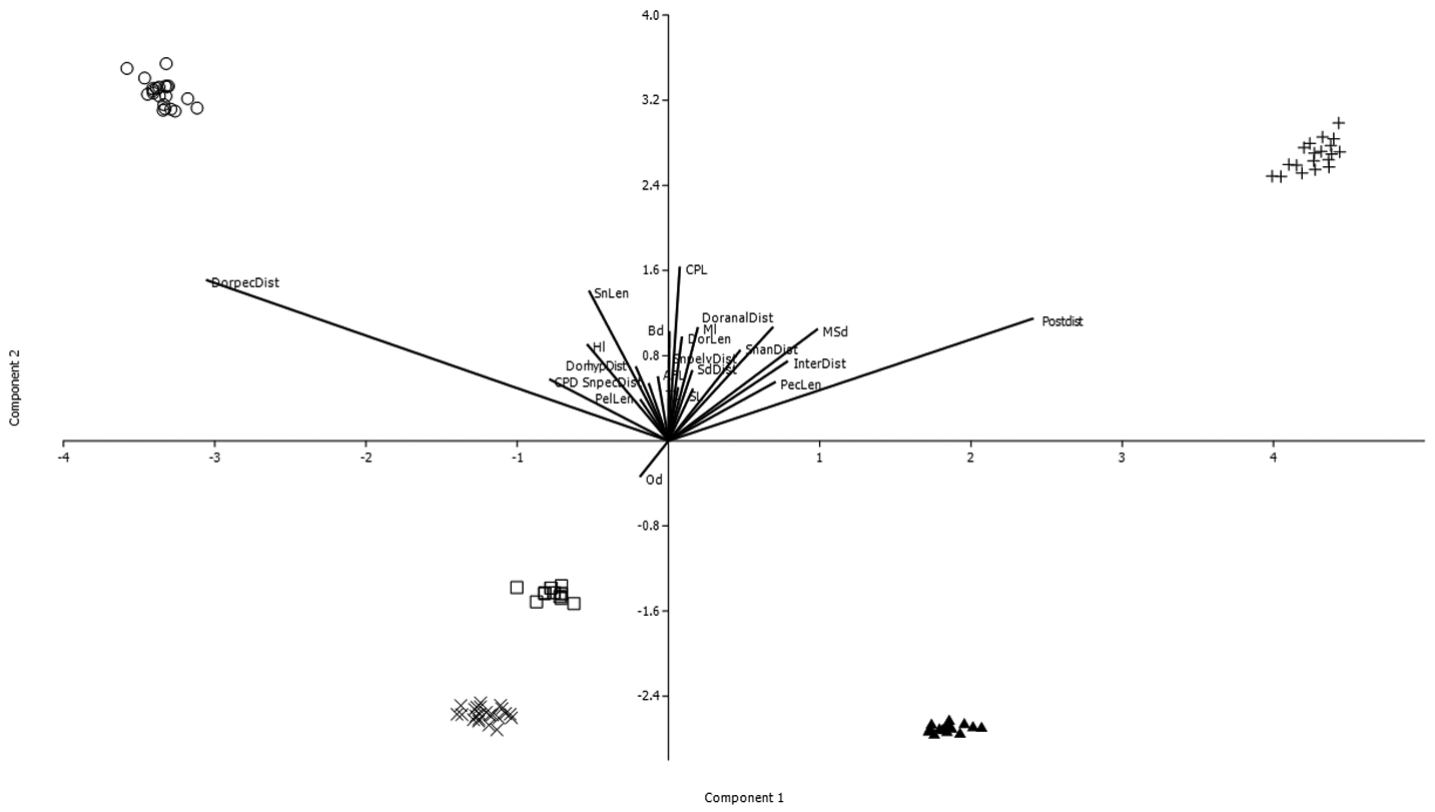
Principal component analysis for morphological data of *Hyphessobrycon klausanni* sp. n. (□), *H. acaciae* (+), *H. mavro* (▲), *H. metae* (○) and *H. niger* (x); component 1 on X axis and component 2 plotted on Y axis. Abbreviations same as Table 1.

Principal component analysis (PCA) detected morphological differences among *Hyphessobrycon klausanni* and *H. acaciae*, *H. mavro*, *H. metae* and *H. niger*. For the first component, upper jaw length, postorbital length and dorsal-pectoral fin distance were the most important variables. For the second component, orbital diameter, caudal peduncle length and snout length were most important. The first component explained 43.71% of total variation, and combined with the second this rose to 85.78% (Fig. 5, Tables 2 and 3).

**Table 2.** Eigenvalue for principal components (PC) between *Hyphessobrycon klausanni* sp. n., *H. acaciae*, *H. mavro*, *H. metae* and *H. niger*.

PC	Eigenvalue	Percentage variance
1	742.872	43.712
2	715.071	42.076
3	143.989	8.4725
4	0.887396	5.2216
5	0.0177261	0.1043
6	0.012861	0.075676
7	0.0106605	0.062728
8	0.00831635	0.048935
9	0.00719985	0.042365
10	0.00544939	0.032065
11	0.00483203	0.028432
12	0.00454893	0.026767
13	0.00389345	0.02291
14	0.00330895	0.01947
15	0.00202874	0.011937
16	0.00152486	0.0089725
17	0.00134239	0.0078988
18	0.00118167	0.0069531
19	0.00103976	0.0061181
20	0.00091383	0.0053771
21	0.000677078	0.003984
22	0.000411058	0.0024187
23	0.000201424	0.0011852

**Table 3.** Eigenvector for first four principal components (PC) between *Hyphessobrycon klausanni* sp. n., *H. acaciae*, *H. mavro*, *H. metae* and *H. niger*. Abbreviations same as Table 1.

	PC 1	PC 2	PC 3	PC 4
SL	0.036926	0.11125	0.053717	0.15823
TL	0.014453	0.11428	0.12973	0.1757
Bd	0.0014034	0.2336	0.19898	0.10276
Sndorsalfin	0.035809	0.15093	0.19889	0.077996
Snpectoralfin	-0.029764	0.12309	0.14127	-0.055979
Snpelvicfin	0.00039923	0.17469	0.19359	-0.035305
Snanalfin	0.10796	0.19393	0.084414	-0.03285
Dorfinhyp	-0.049318	0.15887	0.12493	0.34098
Doranafin	0.020214	0.22269	-0.017732	0.24495
Dorpecfin	0.16087	0.1257	0.18137	0.021098
Dfl	-0.042492	0.088703	-0.12982	0.0055815
Pecfl	-0.17901	0.13165	-0.31013	0.37798
Pelfl	0.016911	0.3717	0.12014	-0.010603
Anfl	-0.12234	0.20629	-0.10574	0.209
CDP	0.15708	0.24308	0.21375	0.093422
CPL	-0.69393	0.34328	-0.0388	-0.34286
HL	-0.016057	0.13764	-0.11393	0.29254
SnL	-0.11936	0.32028	0.22983	-0.24192
ED	-0.043155	-0.076829	0.19662	0.4488
PostOL	0.54766	0.26111	-0.013493	-0.21286
ML	0.044176	0.24261	-0.56997	0.079634
IW	0.17908	0.1701	-0.10416	-0.15303
UJL	0.22414	0.23888	-0.40194	-0.10821

#### Ecological note.

*Hyphessobrycon klausanni* lives in shallow (0.30–1.5 m) well-oxygenated (6.39–7.68 mg/l) streams with transparent waters flowing (0.063 m/s) over different types of substrates (rocks, sand, gravel and decomposing organic material). The temperature range was narrow, 25.5–26°C but pH varied from moderately acidic to basic (6.47–8.7). *Hyphessobrycon klausanni* was found near shore among aquatic vegetation, tree roots and fallen logs. Other species found at the sites included: *Hemigrammus barrigonae*, *Ctenobrycon spilurus*, *Tyttocharax metae*, *Moenkhausia oligolepis*, *M. chrysargyrea*, *M. lepidura*, *Chrysobrycon guahibo*, *Ochmacanthus orinoco*, *Farlowella vittata*, *Ancistrus triradiatus*, *Centromochlus reticulatus* and *Anablepsoides* sp. Stomach content analysis (n = 4) revealed a diet of aquatic and terrestrial invertebrates: Coleoptera (Dytiscidae), Ephemeroptera, Hymenoptera (Formicidae), larvae of Chironomidae and others not identified due to the degree of fragmentation.

## Discussion

Although in our opinion the artificial groupings based on pigmentation patterns proposed by Géry (1977) lack a systematic basis upon which to evaluate relationships among species of *Hyphessobrycon*, some recent contributions use these criteria to segregate groups of species (Lima et al. 2014, García-Alzate et al. 2015, Ohara and Lima 2015, Zarske 2015) in an attempt to clarify the alpha taxonomy that forms the basis of our systematic study of this group. Among these species groups we find those defined by the presence of a dark longitudinal stripe (group e, Géry 1977: 470) which is further subdivided into the *Hyphessobrycon agulha*-group: including species with the lower half of the body all dark, especially above the anal fin, an usually horizontally elongate humeral spot that is more or less united with an asymmetrical, broad lateral stripe (Géry 1977: 470), the *H. agulha*-group was recently defined by Ohara and Lima (2015: 568) to include species that have a broad, relatively diffuse lateral stripe (typically more discernible ventrally, posterior to the midbody) and a humeral blotch that may or may not coalesce with the stripe, included the following species in this group: *H. agulha*, *H. herbertaxelrodi*, *H. metae*, *H. peruvianus*, *H. mutabilis* and *H. loretoensis* (in the latter two, the humeral blotch may or may not coalesce with the stripe), which also includes the following species: *H. clavatus*, *H. lucenorum*, *H. vanzolinii*, *H. margaritae* and *Hyphessobrycon klausanni* sp. n. Also, Lima et al. (2014) define the *Hyphessobrycon heterorhabdus*-group: to include species that share a well-defined, elongated humeral blotch, which is continuous with a midlateral, well-defined dark stripe that becomes blurred towards the caudal peduncle and also have a longitudinal red stripe, extending along the body above the midlateral line, as well as on the upper half of the eye red in living specimens that includes: *H. heterorhabdus*, *H. amapaensis*, *H. eschwartzae* and *H. montagi*. *Hyphessobrycon klausanni* is similar to *H. metae*, with which it shares a well-developed broad midlateral stripe and a well-developed humeral blotch. As mentioned by Ohara and Lima (2015), broad phylogenetic studies on the genus and related genera are necessary to evaluate its putative relationships, but these two species groups could be a starting point. As mentioned in the introduction, the genus *Hyphessobrycon* is paraphyletic and the new species described in this paper is not a member of the genus *Hyphessobrycon* *sensu stricto* (García–Alzate et al. 2013 a, b) which includes the type species *H. compressus* and related species in Central America and some from the Colombian Chocó region: *H. colombianus*, *H. sebastiani*, *H. condotensis*, *H. chocoensis* and *H. ecuadorensis*. A phylogenetic reconstruction is needed that includes the type species and related Central American species to begin to unravel the phylogenetic relationships of the species of *Hyphessobrycon* *sensu lato.*

Rapid taxonomic description of the many as yet unnamed fish species of the Neotropical ichthyological biodiversity is urgently needed given the accelerated rate of extirpation caused by human impacts in many aquatic ecosystems in the Orinoco River Basin. The loss of habitat for fish species is caused by many different human activities such as dam construction, urban water pollution, mining, poor agricultural and animal husbandry practices, the introduction of non-native species and overfishing (Barletta et al. 2012). The Guaviare River drainage in Colombia is an area of high priority for conservation (Machado-Allison et al. 2010), and even with greater recent efforts to sample this system, our knowledge of its fish fauna remains poor, categorized as medium (30–38%) by Portocarrero-Aya et al. (2014). In this contribution a new species, *Hyphessobrycon klausanni*, is described which shows that new taxa will continue to be discovered as more and better inventories are carried out in the upper Guaviare. This also confirms that the Guaviare River drainage remains a priority area for conservation and ichthyological exploration, with regards to species richness and endemism. However, the streams of the upper Guaviare are being impacted by water extraction, and conversion of riparian forests into cattle pastures and monoculture crops such as oil palms. These impacts will undoubtedly be reflected as changes in the composition, richness and ecosystem functions of the fish community and in some cases may eventually lead to the extinction of some species before they have even been discovered or described.

## Supplementary Material

XML Treatment for Hyphessobrycon klausanni
